# Cytokines in aqueous humor of patients with congenital cataract during delayed sequential bilateral cataract surgery

**DOI:** 10.1186/s12886-023-03239-y

**Published:** 2023-11-30

**Authors:** Na Hui, Lei Yu, Laiqiang Qu, Hong Yan

**Affiliations:** Shaanxi Eye Hospital, Xi’an People’s Hospital (Xi’an Fourth Hospital), Affiliated People’s Hospital of Northwest University, 710004 Xi’an, China

**Keywords:** Congenital cataract, Cytokines, Delayed sequential bilateral cataract Surgery

## Abstract

**Background:**

To explore the changes of cytokines expression in aqueous humor (AH) of eyes of patients with congenital cataract (CC) who underwent delayed sequential bilateral cataract surgery (DSBCS).

**Methods:**

28 patients with CC underwent DSBCS. AH samples were collected from each eye before surgery. The contents of cytokines in AH were detected by Luminex xMAP Technology.

**Results:**

There was no significant difference in the expression of IL-8, IP-10, MCP-1 and PDGFAA in the AH of the first and second eyes (*P* = 0.35, 0.39, 0.17, respectively). The level of IL-8 in the first-eye AH was negatively correlated with age (ρ=– 0.519, *P* = 0.008). IP-10 and MCP-1 in the second-eye AH were negatively correlated with age (ρ=– 0.483, *P* = 0.009; ρ=– 0.445, *P* = 0.018,respectively).

**Conclusion:**

The first-eye surgery in patients with CC may not cause the change of cytokines in the contralateral eye. The expression of IL-8, IP-10 and MCP-1 in the AH was negatively correlated with the age of patients.

**Trial registration:**

The study was registered at www.chictr.org.cn on March 22, 2022 and the clinical trial number is ChiCTR2200057927.

## Background

Congenital cataract (CC) is an opacification of crystalline lens detected at birth or within the first decade of life [1]. Since the opacification occurs in infants or young children, lens opacity in optic axis can cause serious visual impairment and even blindness. A meta-analysis including 27 studies showed that the global prevalence of CC was estimated to be 4.24 per 10,000 people worldwide, with the highest prevalence in Asia, estimated at 7.43 per 10,000 people [2]. In terms of prevalence, CC is a rare disease [3], however, it is the most important cause of treatable blindness in infants and children [4].

CC is unilateral or bilateral. In a clinical study of 351 children with CC in Nepal, the bilateral cataract accounted for 43.9% [5]. Another study on CC patients younger than 12 months in South India showed that 77.9% of the children had bilateral cataracts [6]. In the research of Chinese doctors, bilateral cataracts were found in 128 children (128/239, 53.6%) [7]. A systematic review and meta-analysis found that bilateral cataracts accounted for 54.1% in CC [2]. These findings indicate at least half of children with CC had bilateral cataracts.

Cataract surgery in children requires general anesthesia. Immediate Sequential Bilateral Cataract Surgery (ISBCS) can reduce the risks and complications of general anesthesia [8–10]. Another advantage of ISBCS for children is the rapid visual rehabilitation [11] for both eyes to reduce amblyopia in the second eye. Bilateral cataract surgery needing general anesthesia is an indication of ISBCS [9, 12, 13]. However, due to the concern about the potential risk of severe bilateral endophthalmitis, ISBCS for cataract surgery in children is still needs to be treated with caution [10].

As far as we know, the clinical studies on immediate versus delayed sequential bilateral cataract surgery (DSBCS) in children mainly focus on complications [8, 10], anesthesia time [8] and follow-up visits [10]. Few studies concerned about the difference of cytokines between the first eye and the second eye cataract surgeries in children. Our previous and other studies focused on alteration of some cytokines in aqueous humor(AH) during the first and the second eye cataract surgery in adults [14–17]. We have found that TGF-β2 levels in the aqueous humor are elevated in the second eye of high myopia and age-related cataract within short period after sequential cataract surgery [17, 18]. However, research on cytokines in AH of children is far less than that of adults. Wu [19] et al. found that 15 cytokines expressed in AH of patients with CC were different from that of patients with age-related cataract (ARC). Another study showed the cytokines with increased expression in AH of patients after congenital cataract surgery were interferon-inducible protein-10 (IP-10)/CXCL10, monocyte chemoattractant protein-1 (MCP-1)/CCL2, and interleukin 2(IL-2), respectively [20]. Therefore, cytokines in aqueous humor of patients with congenital cataract during delayed sequential bilateral cataract surgery is our motivation and curiosity for further study.

Based on our previous studies in high myopia and age-related cataract eyes in adult, we compared the content of cytokines in AH of patients with CC between the first and the second eye during delayed sequential bilateral cataract surgery.

## Methods

### Patients

This prospective study conducted in Shaanxi Eye Hospital, Xi’an People’s Hospital (Xi’an Fourth Hospital), Xi’an, China, from April 2022 to August 2022. It was approved by the ethics committee of Xi’an People’s Hospital and adhered to the tenets of the Declaration of Helsinki and its amendments. Guardians of each child were informed and signed consent before enrollment. The study was registered at www.chictr.org.cnon22/03/2022 and the clinical trial number is ChiCTR2200057927.

28 patients with bilateral CC were included in this study. Previous history of ocular surgery, other congenital ocular or systemic diseases, including but not limited to glaucoma, corneal disorders, vitreous and retinal diseases, congenital heart disease, with AH content less than 0.5 ml, were excluded from this study. 28 patients with CC underwent lens aspiration, posterior capsulotomy and anterior vitrectomy. Intraocular lens (IOL) were implanted in patients over 2 years old, but not in patients under 2 years old. One eye was randomly selected for the first surgery, which was the first eye group. The other eyes underwent the second surgery after a period of time, which was the second eye group.

All patients underwent thorough ophthalmologic examination, including slit-lamp examination, intraocular pressure, fundus examination and B-scan ultrasonography. Corneal endothelium and IOL master (Carl Zeiss Meditec, Jena, Germany) were examined in patients over 2 years old. The IOL power was calculated by Barrett II formula and the target diopter was determined according to the ocular axis and age of the patient. All surgeries were performed under general anesthesia by the same pediatric cataract surgeon, using a Stellaris platform (Bausch & Lomb Incorporated, Rochester, NY, USA). The procedures included conjunctival peritomy and 2.2 mm corneoscleral tunnel incision at the 11 o’clock position, the anterior continuous circular capsulorhexis with a diameter of about 5.5 mm, lens aspiration, posterior capsulotomy, limited anterior vitrectomy, in-the-bag implantation of IOL MI60 (Bausch & Lomb Incorporated, Rochester, NY, USA) and suturing conjunctival and corneoscleral incision. Postoperatively, a topical treatment included antibiotics (0.3% tobramycin eye drop, 4 times per day for a month) and steroids (1% prednisolone acetate eye drop, 4 times a day for a month).

### Sample collection

Samples of AH (50–200µL) were collected with a 27-gauge needle before any incision was made during each operation. Avoid touching the iris, the corneal endothelium or the lens when the needle entered the anterior chamber from the limbus. AH samples were stored at -80℃ instantly until analysis.

### Analysis of cytokines

The following cytokines including Granulocyte colony stimulating factor (G-CSF), interleukin 1 alpha (IL-1 α), interleukin 6 (IL-6), interleukin 7 (IL-7), epidermal growth factor (EGF), interleukin 3 (IL-3), interleukin 8(IL-8)/CXCL8, MCP-1/CCL2, tumor necrosis factorα(TNFα), platelet-derived growth factor AA (PDGF-AA), IP-10/CXCL10 and IL-2 were measured using a Luminex system (Luminex xMAP Technology, Bio-Rad Laboratories, Inc., Hercules, CA, USA) with Human Premixed Multi-Analyte Kit (Cat:LXSAHM-13, Lot: L141227;R&D system, Minneapolis, MN, USA). All assays were performed in accordance with the manufacturer’s guidelines.

### Statistical methods

Statistical analysis was conducted with SPSS software for Windows (version 23.0, IBM Corp., CA, USA). The detection rate for each cytokine was compared between the first-eye and second-eye group with Fisher’s exact probability test. If the detection rate of any cytokines was more than 85%, the cytokines would be further statistically analyzed. The normality of all data was evaluated with Shapiro-Wilk test. If normality was achieved, student’s t test for paired data and student’s t test for unpaired data were used. If normality was not achieved, Wilcoxon rank-sum test was applied to analyze the paired data, and Mann-Whitney U test was used to compare the data between groups. Spearman’s correlation coefficient was used to analyze the correlation between age, interval and level of cytokines. A *P* value of less than 0.05 was considered as a statistically significant difference for all statistical tests. The results are expressed as the mean ± standard deviation.

## Results

The study included 56 eyes of 28 patients. Both eyes of the same patient were divided into two groups according to the sequencer of surgery: first eye and second eye, so there was no significant difference in age and sex between the two groups. Demographic characteristics of patients was summarized in Table 1. The surgical interval between sequential bilateral cataract surgery was listed in Table 2. The distance between provinces and cities in China is relatively far. In addition, due to the impact of the COVID-19, the span of two children was too large. The mean surgical interval between sequential bilateral cataract surgery was 16.64 ± 22.88 days.

**Table 1:** Patients demographic data

Number	28
Age, mean (SD), years	4.09 ± 2.87 (0.5 to 13)
Sex, n (%)	
Male	17(61%)
Female	11(39%)
Cataract Morphology,n (%)	
nuclear cataract	18 (32.2%)
perinuclear cataract	14 (25%)
total cataract	11(19.6%)
Posterior subcapsular	6 (10.7%)
Mixed or others	7 (12.5%)

**Table 2:** Distribution of interval between SBCS

Interval between SBCS(days )	Number
3	2
4	3
7	9
9	1
10	2
11	3
14	1
17	2
25	1
32	1
33	1
85	1
100	1

12 cytokines were detected in this study, and only 3 cytokines had a detection rate of 100% in AH samples of both eyes. One cytokine had a detection rate of more than 85% in AH samples of both eyes, and the detection rate of the remaining 8 cytokines was not high (Table 3).

**Table 3:** Detection rate of each cytokine in both first- and second-eye AH samples

Cytokines(pg/ml)	Working range(pg/ml)	No. (%) detectable		*P* value
		First-eye	Second-eye	
G-CSF	8.31–6060.00	5/28(17.86%)	4/28(21.43%)	1.000
IL-1 alpha	1.88–1370.00	11/28(39.29%)	9/28(32.14)	0.781
IL-6	1.36–990.00	7/28(25%)	7/28(25%)	1.000
IL-7	1.56–1140.00	19/28(67.86%)	20/28(71.43%)	1.000
TNF-alpha	2.44–1780.00	5/28(17.86%)	4/28(14.29%)	1.000
IL-3	30.07–21920.00	10/28(35.71%)	9/28(32.14%)	1.000
IL-8/CXCL8	1.20–950.0	25/28(89.29%)	26/28(92.86%)	1.000
IP-10/CXCL10	0.55–400.00	28/28 (100%)	28/28 (100%)	1.000
EGF	2.54–1850.00	3/28 (10.71%)	4/28 (14.29%)	1.000
IL-2	9.09–6630.00	7/28(25%)	6/28(21.43%)	1.000
PDGF-AA	1.51–1100.00	28/28 (100%)	28/28 (100%)	1.000
MCP-1/CCL2	9.53–6950.00	28/28 (100%)	28/28 (100%)	1.000

**Fig. 1:**
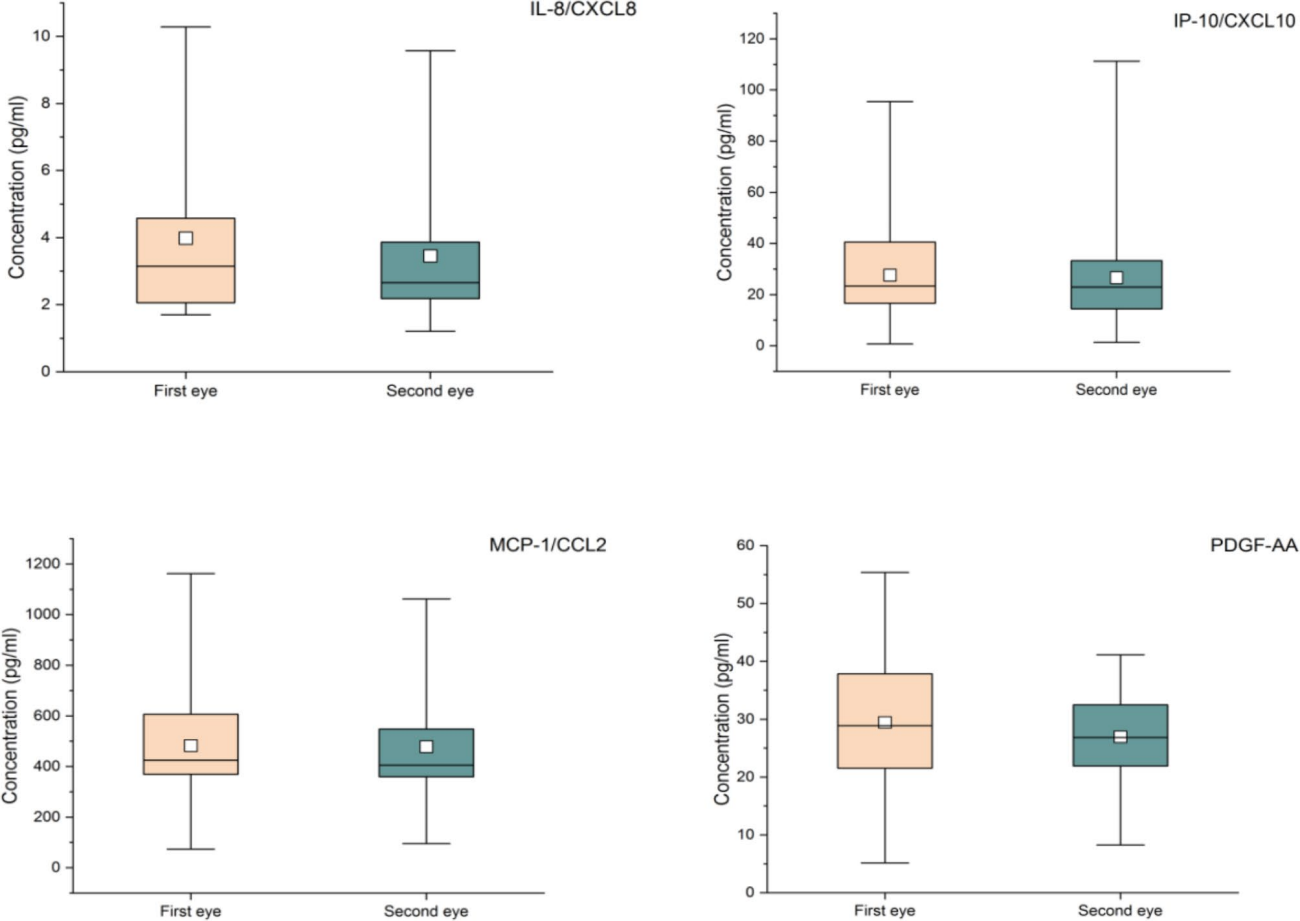
Box-plot of cytokines in AH of patients with congenital cataract

**Fig. 2:**
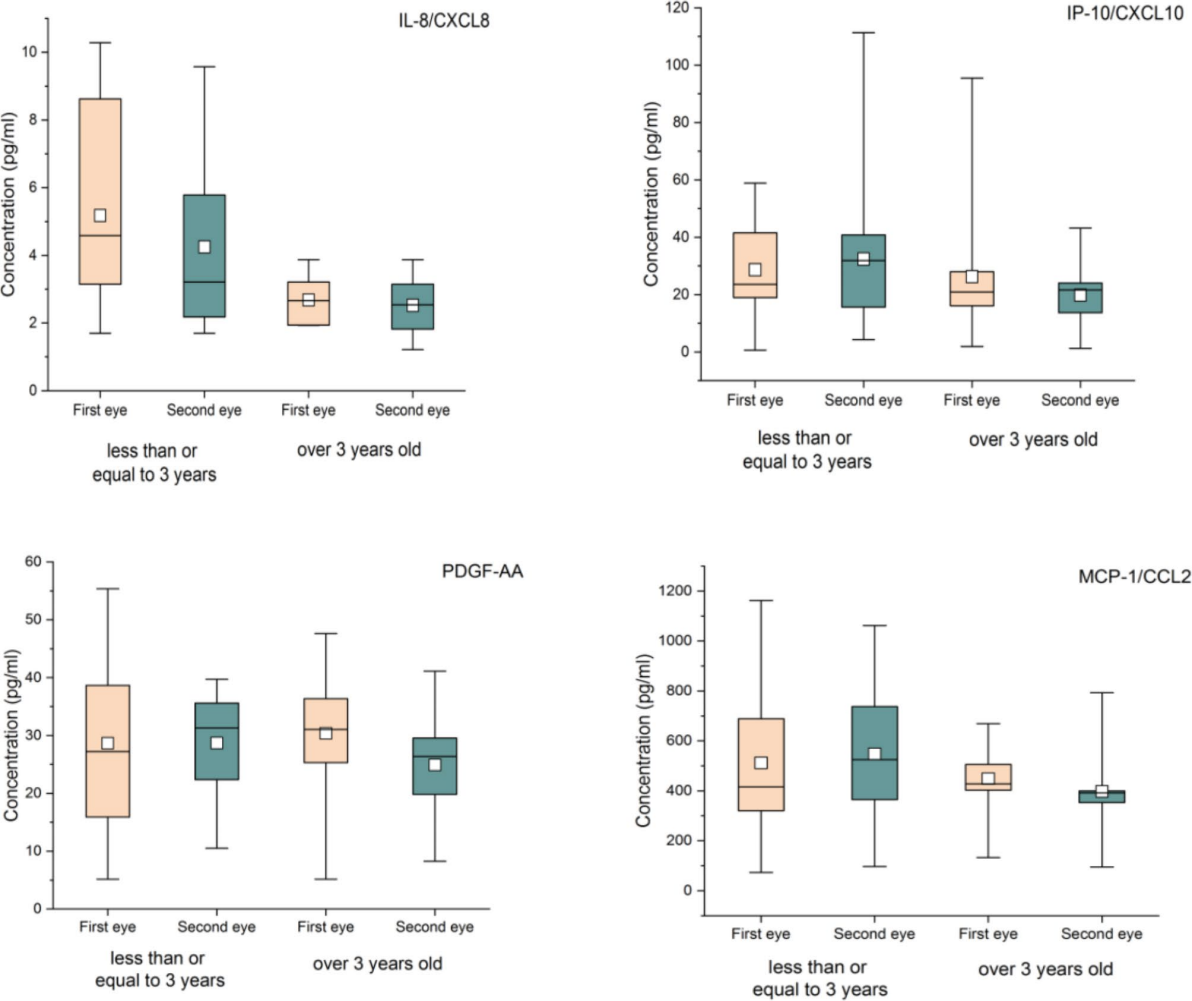
Box-plot of cytokines in first-eye and second-eye AH samples of patients with different ages

**Fig. 3:**
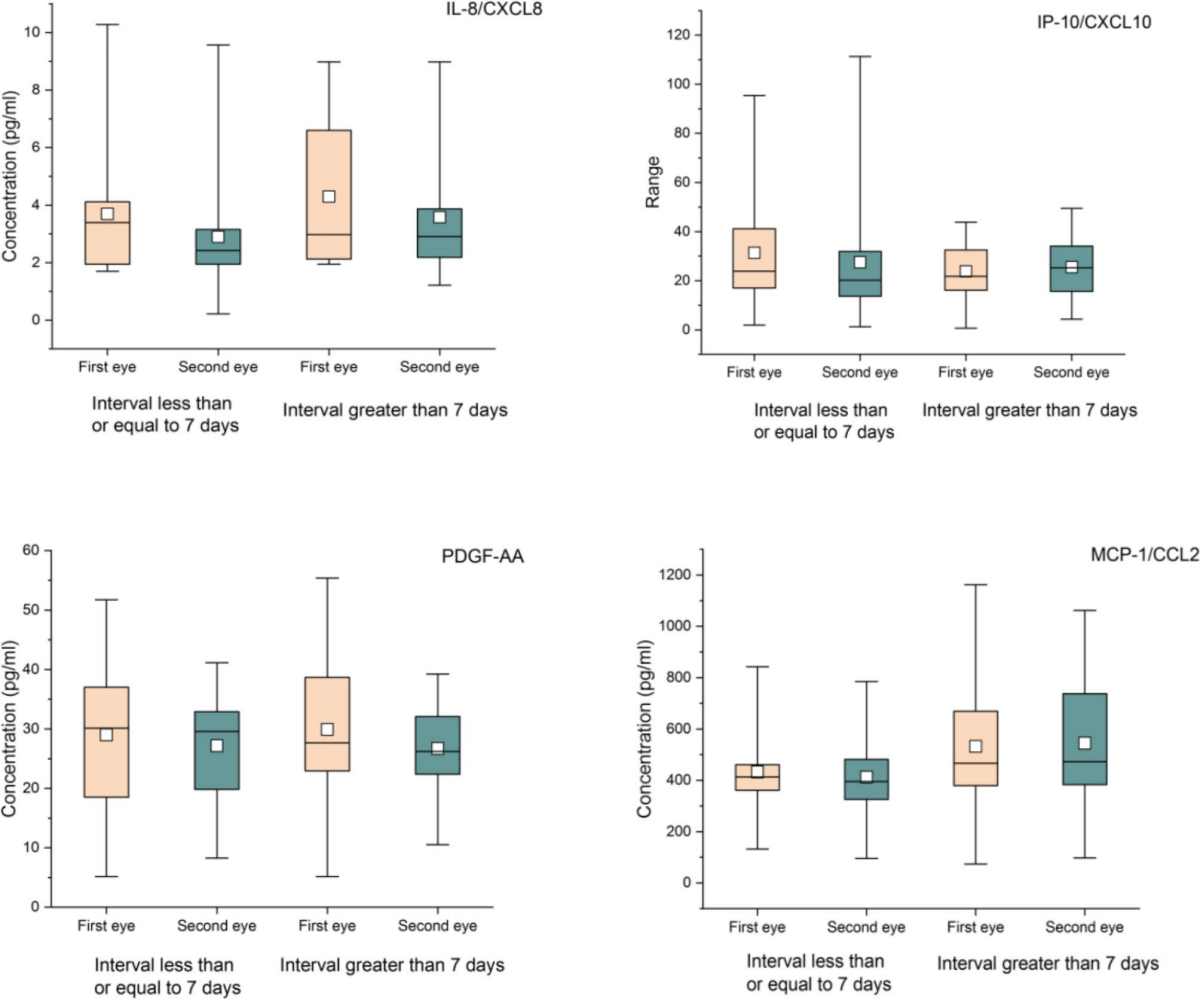
Box-plot of cytokines in first-eye and second-eye AH samples of patients with different intervals

There were no significant differences in IL-8, IP-10, PDGF-AA and MCP-1 in the AH samples of the first eye and the second eye (Table 4; Fig. 1). No significant difference was found in the above 4 cytokines between the first-eye and second-eye AH samples of patients less than or equal to three years old and patients older than three years old (Table 5; Fig. 2), and no significant difference was found in the patients with interval less than or equal to 7 days and with interval greater than 7 days (Table 6; Fig. 3).

**Table 4:** Comparison of cytokines in AH of patients with congenital cataract

Cytokines	Detectable samples, n/n (%) Concentration [M (P25,P75)], pg/ml); Range(pg/ml)		*Z* (Wilcoxon rank-sum test)	*P* value
	First-eye	Second-eye		
IL-8/CXCL8	25/28(89.29%) 3.98(2.00,4.58); 1.7 to 10.28	26/28(92.86%) 3.45(2.12,3.86) 1.21 to 9.57	275.00 (Mann-Whitney U test)	0.35
IP-10/CXCL10	28/28 (100%) 27.57(16.34,40.79) 0.66 to 95.44	28/28 (100%) 26.55(14.07,33.67) 1.29 to 111.31	-0.87	0.39
PDGF-AA	28/28 (100%) 29.45(20.83,38.26) 5.16 to 55.36	28/28 (100%) 26.94(21.65,32.68) 8.24 to 41.13	-1.39	0.17
MCP-1/CCL2	28/28 (100%) 482.35(365.21,606.53) 73.63 to 1162.31	28/28 (100%) 478.55(356.31,555.26) 95.34 to 1062.40	-1.16	0.25

**Table 5:** cytokines in first-eye and second-eye AH samples of patients with different ages

Cytokines	Age less than or equal to 3 years (N = 15) Concentration[M (P25,P75)], pg/ml)				Age over 3 years old (N = 13) Concentration[M (P25,P75)], pg/ml)			
	First-eye	Second-eye	*U*	*P* value	First-eye	Second-eye	*U*	*P* value
IL-8/CXCL8	5.18(2.67,8.69) (N = 13)	4.25(2.18,6.37) (N = 14)	72.5	0.375	2.68(1.94,3.30) (N = 12)	2.52(1.76,3.15) (N = 12)	64	0.67
IP-10/CXCL10	28.68(19.00,41.50)	32.39(15.61,40.79)	108.50	0.87	26.29(13.63,30.27)	19.82(11.63,25,21)	71	0.51
PDGF-AA	28.65(15.89,38.67)	28.69(22.37,35.58)	118.00	0.84	30.37(25.04,37.64)	24.92(18.13,30.26)	56.00	0.15
MCP-1/CCL2	511.52(320.81,688.91)	548.26(365.17,737.40)	127.00	0.57	448.70(390.92,553.49)	398.11(331.70,431.14)	52.00	0.101

**Table 6:** cytokines in first-eye and second-eye AH samples of patients with different intervals

Cytokines	Interval less than or equal to 7 days (N = 14) Concentration[M (P25,P75)], pg/ml)				Interval greater than 7 days (N = 14) Concentration[M (P25,P75)], pg/ml)			
	First-eye	Second-eye	*U*	*P* value	First-eye	Second-eye	*U*	*P* value
IL-8/CXCL8	3.69(1.94,4.35) (N = 13)	2.89(1.82,3.15) (N = 13)	56.00	0.15	4.29(2.09,7.61) (N = 12)	3.58(2.06,3.87) (N = 13)	67.00	0.57
IP-10/CXCL10	31.35(16.39,41.22)	27.56(13.68,34.52)	80.00	0.43	23.79(14.94,34.40)	25.54(14.10,34.25)	105.00	0.52
PDGF-AA	29.00(17.87,37.49)	27.17(19.25,33.56)	89.00	0.70	29.89(22.23,39.53)	26.70(22.13,32.70)	83.50	0.51
MCP-1/CCL2	432.44(351.26,495.89)	412.23(321.47,491.93)	90.00	0.73	532.27(373.09,673.94)	544.87(375.47,751.16)	98.00	1.00

**Fig. 4:**
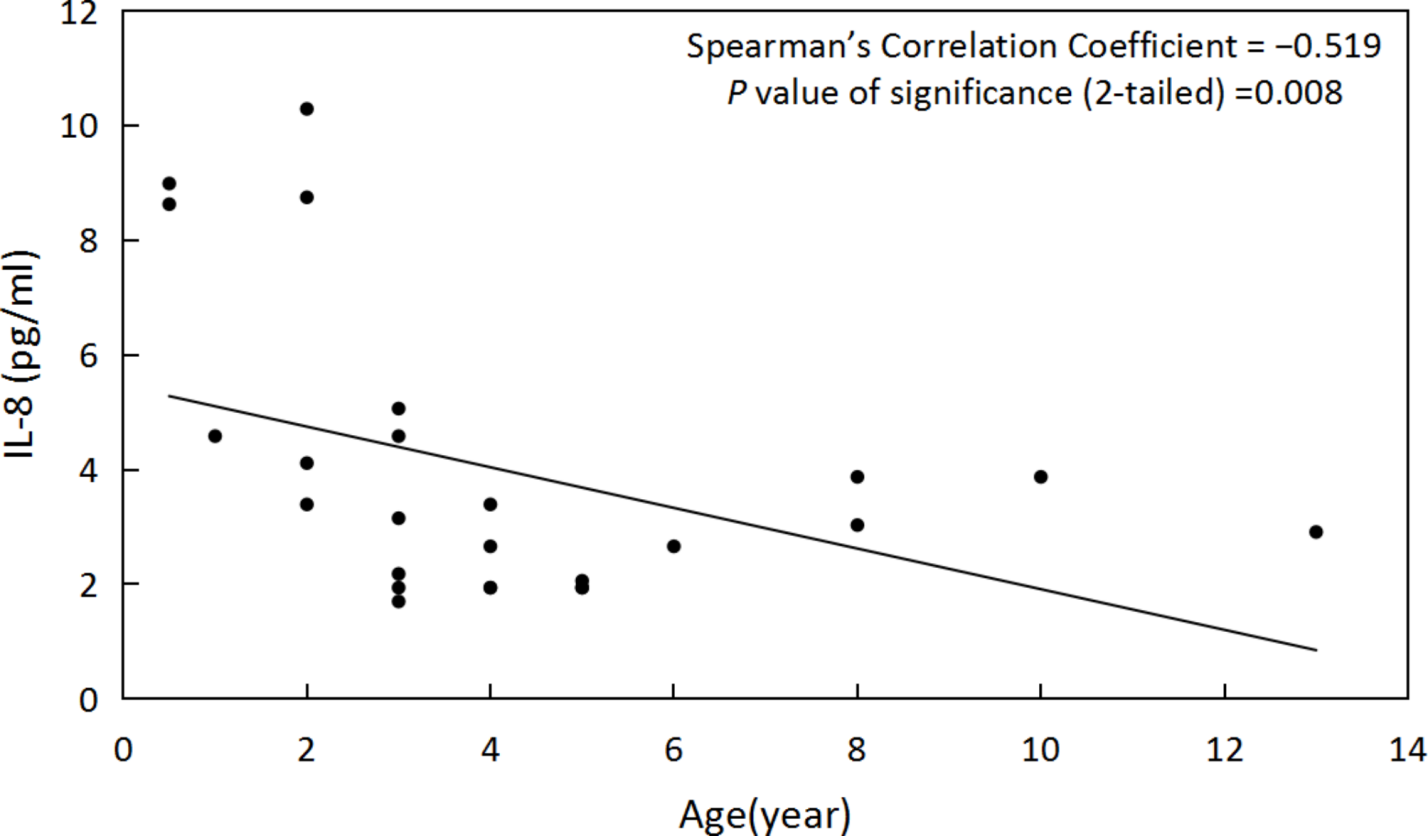
Correlation of IL-8 with age in first-eye AH samples

**Fig. 5:**
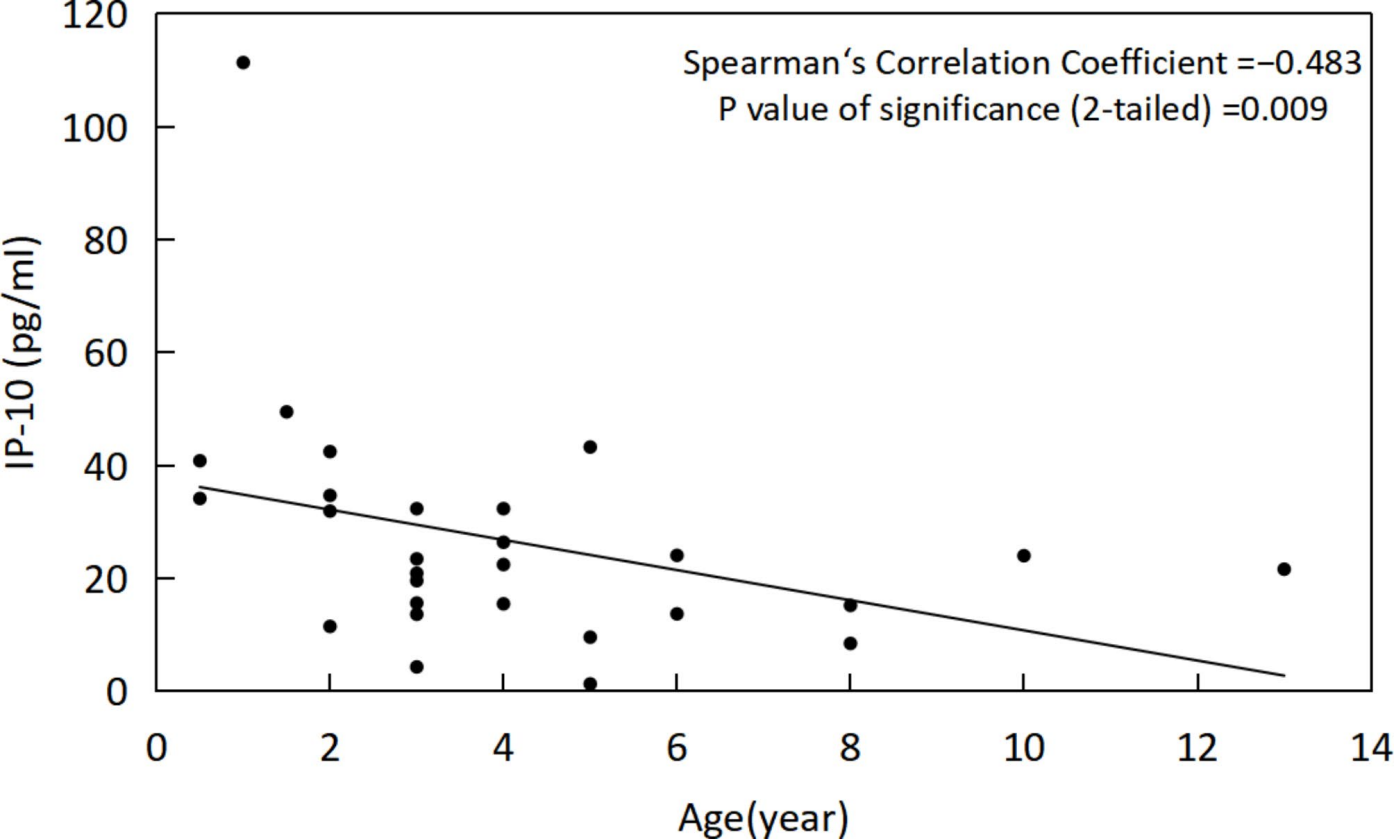
Correlation of IP-10 with age in second-eye AH samples

**Fig. 6:**
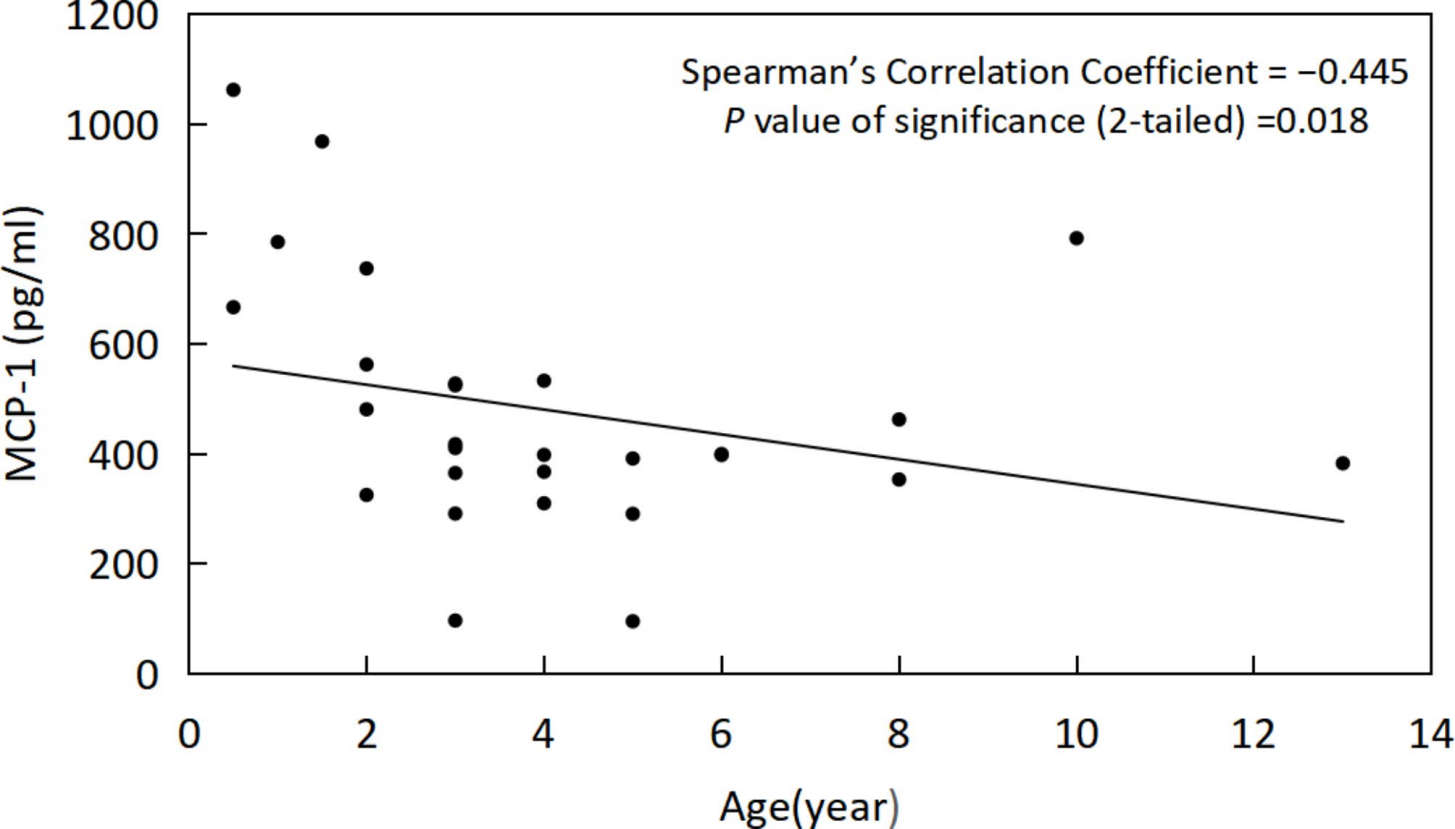
Correlation of MCP-1 with age in second-eye AH samples

In first-eye AH samples, we found that IL-8 was negatively correlated with age (ρ=– 0.519, *P* = 0.008) (Fig. 4), PDGF-AA, IP-10 and MCP-1 had no correlation with age (*P* = 0.946,0.075,0.453, respectively). In second-eye AH samples, IP-10 (ρ=– 0.483, *P* = 0.009) (Fig. 5) and MCP-1 (ρ=– 0.445, *P* = 0.018) (Fig. 6)were negatively correlated with age. IL-8 and PDGF-AA were not correlated with age (*P* = 0.051, 0.06, respectively). IL-8, PDGF-AA, IP-10 and MCP-1 were not correlated with interval (*P* = 0.978, 0.729, 0.417, 0.295, respectively).

## Discussion

To our knowledge, this study is the first to explore the cytokines in AH of the first eye and the second eye of patients with CC during DSBCS. The detection rates of IL-1, IL-2, IL-3, IL-6, IL-7, G-CSF, TNFαand EGF in bilateral AH were no more than 40%. The detection rates of IP-10, MCP-1 and PDGF-AA in the AH of the first eye and the second eye were 100%, and the detection rates of IL-8 were 89.29% and 92.86%, respectively. Previously, we used Luminex system to detect the AH in the first and second eyes of patients with ARC undergoing DSBCS, and also found that the detection rates of IL-1, IL-2, IL-6 and TNFαwere less than 40% [17]. The detection rates of IP-10 and MCP-1 were also 100%, while the detection rate of IL-8 was 88% and 100%, respectively [17]. Therefore, we can speculate that some cytokines are not fully expressed in the AH of patients with CC or ARC, while IP-10 and MCP-1 are 100% expressed.

We compared the contents of IL-8, IP-10, MCP-1 and PDGFAA in the AH of the first eye and the second eye of patients with CC, and the results showed no significant statistical difference. Then we grouped the patients according to age and interval. The contents of the above four cytokines were still not statistically different. IL-8, IP-10, MCP-1 and PDGFAA in the AH of the contralateral eye may not varied in response to the first-eye surgery of patients with CC. Zhao et al. [20] showed that the levels of IL-6, IP-10, MCP-1 and IL-2 in the AH of the second eye were significantly higher than those of the first eye in CC patients. Unlike our study, they compared 23 aphakic eyes with 26 eyes without cataract surgery instead of those with delayed sequential bilateral cataract surgery. This may be the reason why their results are inconsistent with ours.

Our previous study found that IL-8, IP-10 and MCP-1 had no significant difference in the AH of both eyes of ARC patients undergoing sequential cataract surgery [17]. Gong et al. found that MCP-1 and IL-8 had no difference in the AH between the first eye and the second eye of ARC patients without diabetes [16]. In addition, Gong et al. confirmed that MCP-1 and substance P were significantly increased in the second eye of ARC patients with diabetes. It may be that hyperglycemia increases the expression of MCP-1 and substance P and partly explains why ARC patients with diabetes are more likely to feel pain during the second-eye surgery than ARC patients without diabetes [16]. Zhang et al. used the enzyme linked immunosorbent assay (ELISA) kit to detect the AH of 141 patients with bilateral ARC. When the interval was 1 week or 6 weeks, the concentration of MCP-1 in the second eye was higher than that in the first eye [14]. However, 32 of these 141 ARC patients were diabetes, which may affect the research results. Zhu et al. compared the cytokines in the AH of ARC patients with an interval of 1 month, and found that the concentration of MCP-1 of the second eye was significantly higher than that of the first eye [21]. Instead of comparing the first eye and the second eye of the same patient, they compared some patients who had the first eye surgery with other patients who had the second eye surgery.

Previous studies have found that IL-8 and MCP-1 in the AH of CC patients without surgery are negatively related to age [19, 22]. We also confirmed that the expression level of IL-8 in the AH of the first eye was negatively related to age. In addition, the expression of IP-10 and MCP-1 in the AH of the contralateral eye after the first-eye surgery was negatively correlated with age in this study. IL-8, IP-10 and MCP-1 are chemokines. Under inflammatory conditions, chemokines are produced by immune and other cell types [23]. Any inflammatory process is characterized by the presence of cytokines and chemokines [24]. IL-8, also known as CXCL8, is mainly used to attract neutrophils to participate in acute inflammation [25]. It is also chemotactic to endothelial cells and plays a major role in angiogenesis [25]. IP-10, also known as CXCL10, is a protein that can be induced by interferon to attract lymphocytes. It has been proved that IP-10 can chemotactic monocytes, activated T cells and expandable NK cells [26–27]. MCP-1, also known as CCL2, is one of the key chemokines regulating migration and proliferation of monocytes/macrophages [28]. In theory, these three cytokines may participate in the inflammatory reaction after CC surgery. Due to ethical reasons, we cannot obtain the postoperative AH of CC patients. The negative correlation between the expression of IP-10 and MCP-1 in the contralateral eye and age may explain the clinical phenomenon that the postoperative inflammatory reaction in the eyes of young children is more serious.

The limitations of this study are as follows: First, the sample size is relatively small. Second, some cytokines were not detected in this study due to the limited volume of AH, such as TGF-β. Third, the AH of healthy children cannot be obtained as the control group.

In conclusion, for patients with CC who need general anesthesia, the advantages of ISBCS are obvious. If the first-eye surgery causes changes in cytokines in the AH of the contralateral eye, DSBCS may be a better choice to alleviate the impact of cytokine changes on the contralateral eye. The findings of this study indicates that the first-eye surgery may not lead to the change of cytokines. For children with congenital cataracts, to some extent, DSBCS has no advantage over ISBCS at the level of cytokines.

## Data Availability

The datasets used and analysed during the current study are available from the corresponding author on reasonable request.

## Abbreviations

AH: aqueous humor
CC: congenital cataract
ARC: age-related cataract
DSBCS: delayed sequential bilateral cataract surgery
ISBCS: Immediately Sequential Bilateral Cataract Surgery
IOL: Intraocular lens
TGF-β2: transforming growth factor-β2
IP-10: interferon-inducible protein-10
MCP-1: monocyte chemoattractant protein-1
IL-2: interleukin 2
G- CSF: H- Granulocyte colony stimulating factor
IL-1α: interleukin 1 alpha
IL-6: interleukin 6
IL-7: interleukin 7
IL-3: interleukin 3
IL-8: interleukin 8
EGF: epidermal growth factor
TNFα: tumor necrosis factorα
PDGF-AA: platelet-derived growth factor AA
ELISA: enzyme linked immunosorbent assay
